# Luminescence in Manganese (II)-Doped SrZn_2_S_2_O Crystals From Multiple Energy Conversion

**DOI:** 10.3389/fchem.2020.00752

**Published:** 2020-09-04

**Authors:** Ronghua Ma, Shaohui Mao, Chunfeng Wang, Yonghong Shao, Zhihao Wang, Yu Wang, Sicen Qu, Dengfeng Peng

**Affiliations:** ^1^Key Laboratory of Optoelectronic Devices and Systems of Ministry of Education and Guangdong Province, College of Physics and Optoelectronic Engineering, Shenzhen University, Shenzhen, China; ^2^International Collaborative Laboratory of 2D Materials for Optoelectronics Science and Technology of Ministry of Education, SZU–NUS Collaborative Innovation Center for Optoelectronic Science & Technology, Institute of Microscale Optoelectronics, Shenzhen University, Shenzhen, China; ^3^Department of Physical Education, Shenzhen University, Shenzhen, China

**Keywords:** light emission, mechanoluminescence, multimode luminescence, X-ray, SrZn_2_S_2_O

## Abstract

Under the excitation of ultraviolet, X-ray, and mechanical stress, intense orange luminescence (Mn^2+^, ^4^T_1_ → ^6^A_1_) can be generated in Mn^2+^-doped SrZn_2_S_2_O crystal in orthorhombic space group of Pmn2_1_. Herein, the multiple energy conversion in SrZn_2_S_2_O:Mn^2+^, that is, photoluminescence (PL), X-ray-induced luminescence, and mechanoluminescence, is investigated. Insight in luminescence mechanisms is gained by evaluating the Mn^2+^ concentration effects. Under the excitation of metal-to-ligand charge-transfer transition, the most intense PL is obtained. X-ray-induced luminescence shows similar features with PL excited by band edge UV absorption due to the same valence band to conduction band transition nature. Benefiting much from trap levels introduced by Mn^2+^ impurities, the quenching behavior mechanoluminescence is more like the directly excited PL from Mn^2+^ d-d transitions. Interestingly, this concentration preference leads to varying degrees of spectral redshift in each mode luminescence. Further, SrZn_2_S_2_O:Mn^2+^ exhibits a good linear response to the excitation power, which makes it potential candidates for applications in X-ray radiation detection and mechanical stress sensing.

## Introduction

Luminescent materials could somehow absorb electrical, optical, chemical, thermal, or mechanical energy and turn it into light emission through a radiative transition. In the last centuries, researchers have been working persistently on optimizing of preparation technique, exploring new materials to meet the rising demand for high-performance luminescent materials in applications such as lighting (Meyer and Tappe, 2015; Xu et al., 2016; Zak et al., 2017), display (Withnall et al., 2011; Ballato et al., 2013), sensing (Eliseeva and Bunzli, 2010; Olawale et al., 2011; Wang et al., 2013; Hu et al., 2014), optoelectronics (Li et al., 2015, 2017), and anti-counterfeiting (Zhang et al., 2018a). While pursuing its state-of-the-art materials by realizing one mode of energy transformation, one single material that could efficiently transform multiple types of energy to light emission has entered people's vision. These materials may bring to life many new appealing applications in the interdisciplinary fields (Singh et al., 2011; Liu et al., 2018; Xu et al., 2018; Zhang et al., 2018b, 2020; Jiang et al., 2019; Sang et al., 2019).

During the past decades, mechanoluminescent materials with the capability of converting mechanical energy to light emission are attracting more and more attention for their potential applications in stress sensing, anti-counterfeiting, display, structure fatigue diagnosis, and flexible optoelectronics (Chandra and Chandra, 2011; Jeong et al., 2014; Liu et al., 2019; Wang C. et al., 2019; Wang X. et al., 2019; Zuo et al., 2019; Wang et al., 2020). On the other hand, almost immediately after the discovery of X-rays, people started eagerly to find efficient X-ray phosphors or scintillators that absorb X-ray and emit light (Blasse, 1994; Büchele et al., 2015; Chen et al., 2018; Lian et al., 2020). With a strong ability to absorb X-ray photons, impurity-doped ML materials may also be good scintillators with intense X-ray-induced emission and find their application in X-ray detection. Recently, much effort has been independently made to optimize the performance of ML and X-ray phosphors. Starting from the two classic ML materials, that is, Eu^2+^-doped SrAl_2_O_4_ and Mn^2+^/Cu^2+^-doped ZnS, in the early stage, many impurity-doped systems have been discovered (Peng et al., 2015; Zhang et al., 2019). Among these, some typical oxysulfides notably enriched the color of ML emission due to its good acceptance for a large number of activators (Zhang et al., 2013, 2015). It is proved that some new oxysulfide semiconductors that doped with luminescent ions, for instance, transitional metals and lanthanide ions doped CaZnOS (Huang, 2016; Huang et al., 2017; Du et al., 2019), SrZnOS (Chen et al., 2020), and BaZnOS (Li et al., 2016) single compounds as well as CaZnOS-ZnS heterojunctions (Peng et al., 2020) show novel ML performances that result in diverse applications. Meanwhile, some oxysulfides, such as Gd_2_O_2_S-based luminescent material (Büchele et al., 2015), has been commercialized and confirmed to be good X-ray phosphors. As oxysulfides show a strong ability to absorb high-energy X-ray photons, they may be good scintillators to meet the rising demand for radiation detection materials as well.

SrZn_2_S_2_O, as a newly discovered oxysulfide semiconductor with close-packed corrugated double layers of ZnS_3_O tetrahedron in its crystal structure, was first reported by Hans-Conrad zur Loye's group (Tsujimoto et al., 2018) and found to function as a high-stable photocatalyst capable of reducing and oxidizing water (Nishioka et al., 2019). Deducing from its non-central symmetry crystal structure and appropriate band structure, impurity-doped SrZn_2_S_2_O could have good ML performance, which was already proved recently (Chen et al., 2020). Herein, we report Mn^2+^-doped SrZn_2_S_2_O material could simultaneously respond to UV light or X-ray exposure or mechanical actions with intense orange emission and realize multiple-energy conversions. Besides mechanical stimulation, SrZn_2_S_2_O:Mn^2+^ can also respond to X-ray exposure with strong visible emission. The ML performance is optimized by choosing the optimal preparation condition and tuning the doping concentration. Strong relevance between ML intensity and the applied mechanical force makes SrZn_2_S_2_O:Mn^2+^ a good candidate for dynamic stress visualization. Notably, the differences in quenching behavior provide us a better understanding of the ML process. This multimode energy conversion behavior might be used in manufacturing future sensing devices.

## Experimental

### Materials Preparation

The samples were prepared by using a high-temperature solid-state reaction. To obtain *x* mol% Mn^2+^-doped SrZn_2_S_2_O, that is, SrZn_2(1−x%)_S_2_O:2*x*% Mn^2+^ (*x* = 0, 0.25, 0.5, 0.75, 1, 2, 3, 4, 6, and 8), high purity of ZnS (99.99%, Aladdin), SrCO_3_ (99.9%, Aldrich), and MnCO_3_ (>99%, Sinopharm Group Co. Ltd.) with mole ratio of 2-2*x*%:1:2*x*%, were used as starting materials. A total of 20 g of raw materials was precisely weighed and then thoroughly mixed by wet grinding in absolute ethanol. After dried in an oven at 80°C, the raw materials were calcined at 1,000°C for 4 h in Ar atmosphere (purity, 99.99%). The sintered product was grounded into fine powders for subsequent characterization.

### Mechanoluminescence Film Fabrication

A “suspension deposition” method is applied to fabricate the mechanoluminescence (ML) film for ML test and exhibition. In a typical case, 0.2 g SrZn_2_S_2_O:Mn^2+^ ML powder and 0.06 g UV curing adhesive (LEAFTOP 9307) were ultrasonically dispersed in ethanol, followed by a rapid transfer into a 3 × 3-cm square frame stainless mold placed on one piece of the ethylene-vinyl acetate-covered poly(ethylene terephthalate) film in a laminating film (Deli, no. 3817). The mold was removed after the volatilization of ethanol; then, the two pieces of ethylene-vinyl acetate-covered poly(ethylene terephthalate) films were folded together very carefully. Subsequently, the film was exposed in UV light to solidify the adhesive and then packaged by going through a thermal laminator.

### Characterization

X-ray diffraction (XRD) patterns were recorded by a Bruker D2 phase X-ray diffraction analyzer. Scanning electron microscope images were obtained from a 3 Hitachi SU 8020 scanning electron microscope. Energy-dispersive X-ray element maps were obtained on a HOBIRA EMAX X-ray detector. Photoluminescence (PL) spectra were measured by Hitachi F-4600 spectrophotometer equipped with an R928 photomultiplier detector. The ML emission spectra were recorded by a home-built measuring apparatus with a linear motor, digital push–pull gauge, and QE65pro fiber optic spectrometer (Ocean Optics). The X-ray-induced emission spectra were obtained by Omni-λ 300i spectrograph (Zolix) equipped with an X-ray tube (Model RACA-3, Zolix Instruments Co., Ltd., Beijing, China).

To acquire the ML spectra, we stick the ML film on a quartz glass plate firmly fixed on the table. On the front side, the digital push–pull gauge with a metal attachment is fixed on a platform connected to a linear motor. On the backside, the fiber connected with the QE65pro fiber optic spectrometer is fixed on the same platform over against the metal attachment. The push–pull gauge tunes the acting force of the metal attachment on ML film, and the programmed linear motor controls the movement of the platform. During the test, the motion of metal attachment on ML film generates light emission, whereas the fiber collects the signal synchronously.

## Results and Discussion

**Figure 1:**
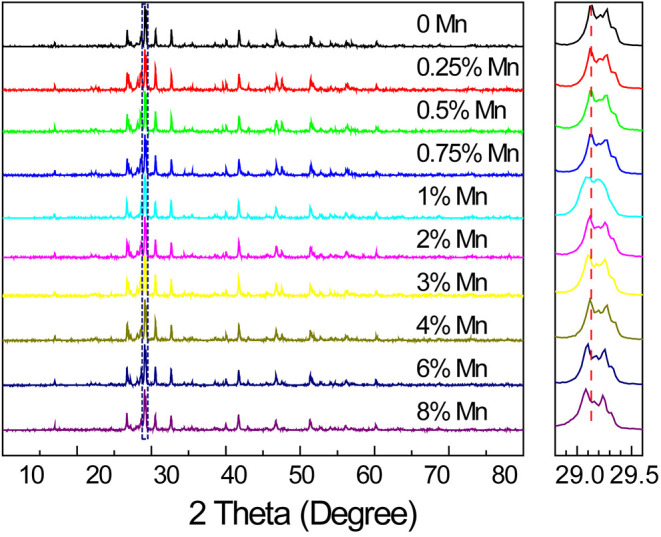
X-ray diffraction patterns of SrZn_2(1−x%)_S_2_O:2*x*% Mn^2+^ with a series of Mn^2+^ concentrations (*x* = 0, 0.25, 0.5, 0.75, 1, 2, 3, 4, 6, and 8), and an enlargement of the highest diffraction peak marked by a dotted rectangle is given in the right side.

We found that 1,000°C was the most suitable temperature for the preparation of SrZn_2_S_2_O:Mn^2+^ to get a good crystallinity while avoiding any decomposition (Supplementary Figure 1). The experimental XRD pattern of the sample calcined at 1,000°C for 4 h matches well with the theoretical calculated powder X-ray diffraction result based on work of Hans-Conrad Tsujimoto et al. (2018), which indicates that the single phase of SrZn_2_S_2_O in orthorhombic space group of Pmn2_1_ (no. 31) was successfully synthesized (Supplementary Figure 2). In the SrZn_2_S_2_O crystal structure, each Zn atom is coordinated with 1 O atom and 3 S atoms as ZnS_3_O tetrahedron, whereas Sr^2+^ ions vertically separate close-packed corrugated double layers of ZnS_3_O tetrahedron. Mn^2+^ will occupy the Zn^2+^ site due to their close radius (Mn^2+^ is slightly larger than Zn^2+^) and similar chemical properties. As a result, the XRD peaks show a slight shift toward lower angle in SrZn_2_S_2_O:Mn^2+^ with increasing Mn^2+^-doping concentration, and there is no second phase found even when Mn^2+^ concentration is rather high (Figure 1). The success in the synthesis of heavily doped SrZn_2_S_2_O:Mn^2+^ makes it easier for further performance tuning to get the desired material performances. After grinding and sifting, we got fine powder several micrometers in size with no regular shape for all the characterization and property tests. Energy-dispersive X-ray spectroscopy verifies the presence of the doped Mn element and its uniform distribution together with Zn, S, Sr, and O elements as a single phase SrZn_2_S_2_O:Mn^2+^ (Supplementary Figure 3).

**Figure 2:**
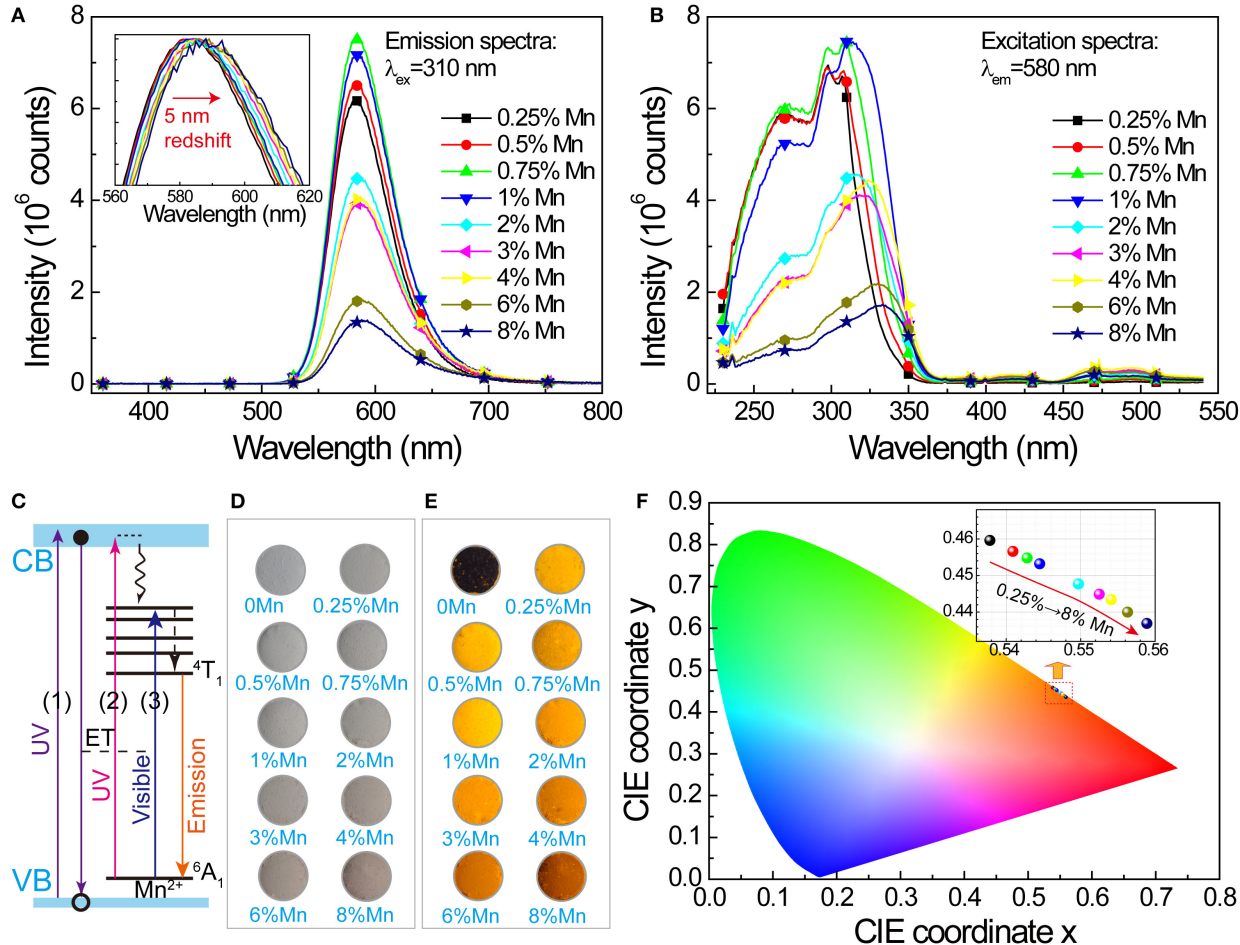
Photoluminescence in SrZn_2(1−x%)_S_2_O:2*x*% Mn^2+^, with *x* = 0.25, 0.5, 0.75, 1, 2, 3, 4, 6, and 8. **(A)** PL spectra under 310 nm excitation. Inset shows a 5-nm redshift of the normalized PL band with increasing doping concentration. **(B)** Corresponding PLE spectra monitoring 580-nm emission. **(C)** Luminescence mechanism of SrZn_2_S_2_O:Mn expressed by energy level diagram. Optical images of the samples: **(D)** under daylight lamp lighting and **(E)** illuminated by 254-nm UV lamp. **(F)** CIE coordinate (x, y) values obtained from the PL spectra under 310-nm excitation; the inset shows the enlargement of the marked area.

PL properties of Mn^2+^-doped SrZn_2_S_2_O are investigated. Under ultraviolet excitation at 310 nm (Figure 2A), a broadband orange emission (~520–700 nm) centered at about 580 nm corresponding to ^4^T_1_ → ^6^A_1_ transition of Mn^2+^ in SrZn_2_S_2_O lattice. With increasing Mn^2+^-doping amount, the emission band shows a slight redshift of about 5 nm and a slightly raising band tail at long wavelength due to reduced energy difference by an exchange interaction effect between two neighboring Mn^2+^ cations (Barthou, 1994; Vink et al., 2001). The emission intensity increases at lower Mn^2+^ concentrations, and then, a fast decrease is witnessed due to the comprehensive effects of excitation efficiency changes and concentration quenching. The corresponding excitation spectra (Figure 2B) comprise a broad UV band including band edge absorption of SrZn_2_S_2_O host at ~270 nm and metal-to-ligand charge transfer (ML_CB_CT) (Norberg et al., 2004; Badaeva et al., 2011) absorption of Mn^2+^ centered at ~320 nm and several smaller bands in the visible light region due to d-d transitions of Mn^2+^. Among these, the UV band, or more precisely, the ML_CB_CT band of Mn^2+^ shows a distinct redshift. Correspondingly, three excitation routes are proposed in the luminescence mechanism depicted in Figure 2C. Namely, route 1 represents the excitonic transition (SrZn_2_S_2_O VB → CB transition), route 2 represents the charge-transfer states transition (Mn^2+^ 3d → CB transition), and route 3 represents d-d transitions of Mn^2+^ (Mn^2+^: ^6^A_1_(^6^S) → ^4^E(^4^D), ^4^T_2_(^4^D), (^4^A_1_,^4^E)(^4^G),^4^T_2_(^4^G) or ^4^T_1_(^4^G)), whereas notably, each excitation routes performs inconsistently with increasing Mn^2+^-doping concentration (Supplementary Figure 4). Because strong concentration quenching of the orange emission occurs when Mn^2+^ concentration is higher than 4% for Mn^2+^ direct excitation (route 3), the concentration effects of routes 1 and 2, with a much lower quenching concentration, should be dominated by the host lattice-related excitation process and the energy transfer process.

Whereas, the undoped SrZn_2_S_2_O is white under daylight lamp lighting, with increasing Mn^2+^-doping concentration, SrZn_2_S_2_O:Mn^2+^ gets darker and darker pink colors (Figure 2D) owing to the slight absorption of blue and green light by Mn^2+^ d-d transitions. Illuminated by a 254-nm UV lamp, the undoped SrZn_2_S_2_O shows no luminescence, whereas varied orange emission is observed in these doped samples (Figure 2E). Figure 2F displays the chromaticity diagram with International Commission on Illumination (CIE) coordinate (x, y) values obtained from the PL spectra under excitation at 310 nm. All chromaticity points locate in the orange region between the yellow and red region, with increasing Mn^2+^ concentration; the CIE coordinate (x, y) varies systematically from (0.538, 0.460) to (0.559, 0.437) due to the redshift of PL. To summarize, SrZn_2_S_2_O:Mn^2+^ realizes light energy conversion, especially, UV to orange light conversion with Mn^2+^ concentration around 1%.

**Figure 3:**
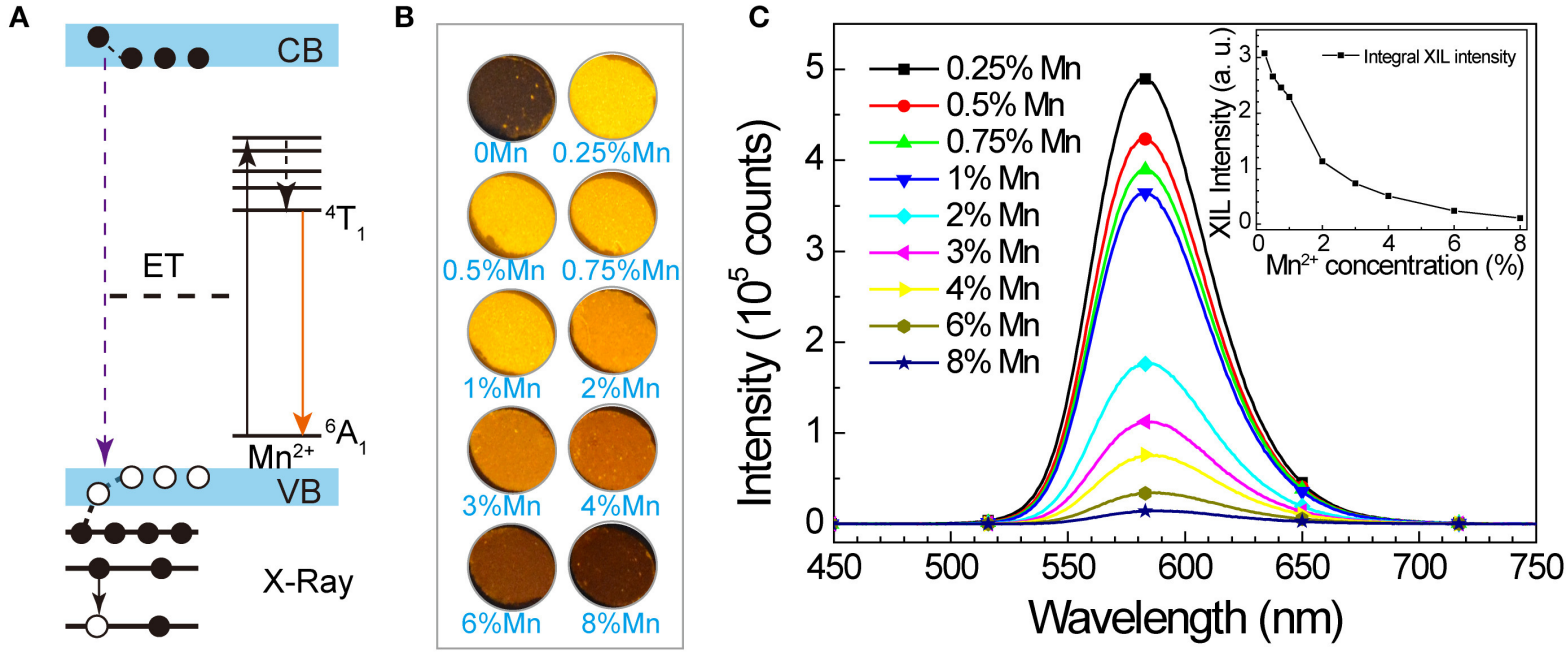
X-ray-induced luminescence in SrZn_2(1−x%)_S_2_O:2*x*% Mn^2+^, with *x* = 0.25, 0.5, 0.75, 1, 2, 3, 4, 6, and 8. **(A)** Scheme of proposed XIL mechanism in SrZn_2_S_2_O:Mn^2+^. **(B)** Optical images of the samples under X-ray irradiation. **(C)** X-ray-induced emission spectra under excitation of X-ray source operating at *U* = 50 kV and *I* = 40 μA. Inset shows the integral intensity in relation to Mn^2+^ concentration.

**Figure 4:**
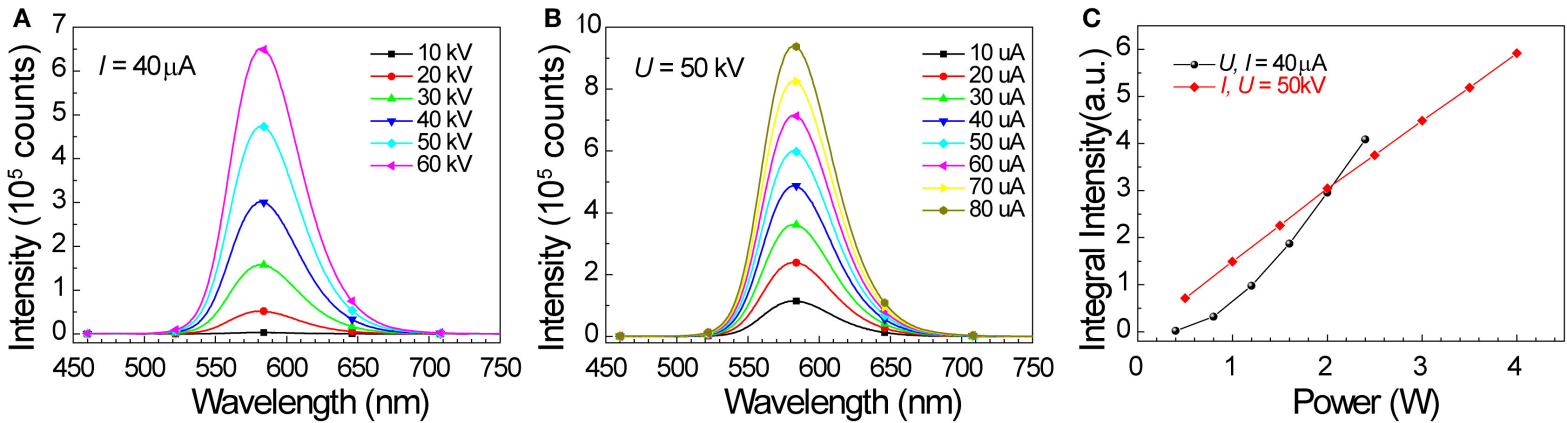
X-ray-induced emission spectra of 0.25% Mn doped SrZn_2_S_2_O under excitation of X-ray source operating at **(A)** *I* = 40 μA, *U* = 10, 20, 30, 40, 50, and 60 kV, and **(B)** *U* = 50 kV, *I* = 10, 20, 30, 40, 50, 60, 70, and 80 μA. **(C)** Integral XIL intensity obtained from XIL spectra in **(A,B)** in relation to operating power (*P* = *UI*).

We also studied the potential of SrZn_2_S_2_O:Mn^2+^ in energy conversion from X-ray to visible light. When SrZn_2_S_2_O is irradiated by X-ray, a large number of electron-hole pairs will be generated mainly via photoelectric effect when absorbing X-ray energy, and later, Mn^2+^ is excited by the energy transferred from the electron-hole pair, followed by an orange light emission process (Figure 3A) (Blasse, 1994; Cao et al., 2016; Teng et al., 2020; Zhang et al., 2020). Observed under X-ray irradiation (Figure 3B, Supplementary Figure 4, and Supplementary Video 1), 0.25% Mn^2+^-doped sample exhibits the most intense orange emission, and the X-ray-induced luminescence (XIL) decreases with increasing Mn^2+^ concentration. This trend is verified by the XIL spectra (Figure 3C) obtained under excitation of X-ray source operating at *U* = 50 kV and *I* = 40 μA, whereas a slight redshift by 3 nm of XIL band is observed. As X-ray irradiation produces electron-hole pairs, which is similar to UV band-edge excitation (PLE route 1), a similar concentration effect is predictable (Supplementary Figure 5). The severe concentration quenching most probably caused by band structure changes of SrZn_2_S_2_O brought by Mn^2+^ doping through the so-called Auger de-excitation effect (White et al., 2011; Peng et al., 2012) resulting in remarkably increased non-radiative transition probability. Lightly Mn^2+^ impurity-doped is favored for SrZn_2_S_2_O:Mn^2+^ to fulfill the X-ray to visible light energy conversion. We further investigated XIL with varied X-ray source operating conditions (Figure 4). When the operating current is fixed at 40 μA (Figure 4A), the integral XIL intensity increased non-linearly with increasing operating voltage, as X-ray photon with higher energy can generate more electron-hole pairs (Scholze et al., 1996). While fixing operating voltage at 50 kV (Figure 4B), the integral XIL intensity is highly proportional to the operating current, which makes SrZn_2_S_2_O:Mn^2+^ material suite well for X-ray detection application.

**Figure 5:**
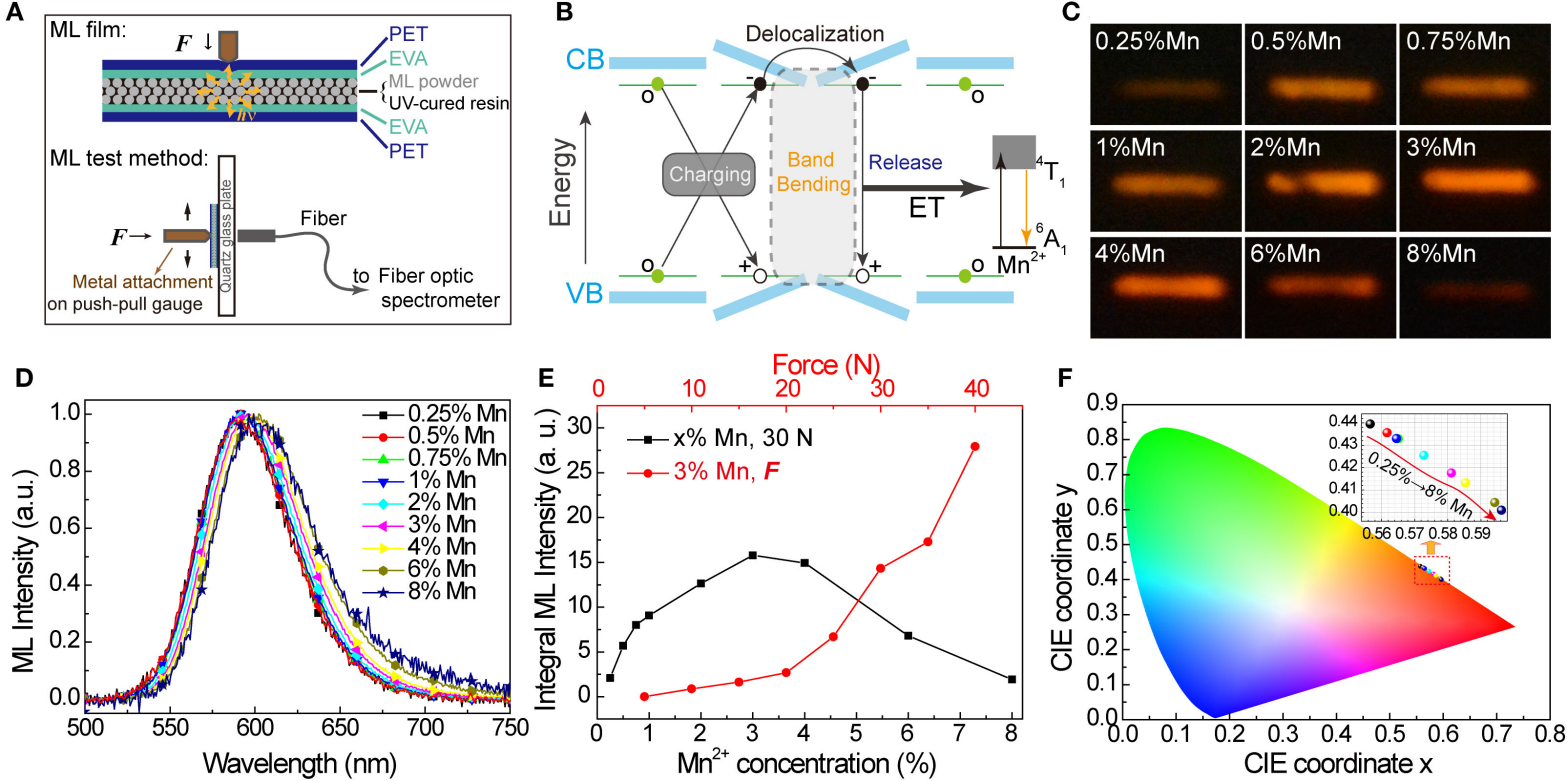
Mechanoluminescence in SrZn_2(1−x%)_S_2_O:2*x*% Mn^2+^, with *x* = 0.25, 0.5, 0.75, 1, 2, 3, 4, 6, and 8. **(A)** Schematic diagram of the ML film structure and ML test method. **(B)** Scheme of proposed mechanism for mechanical-to-optical energy conversion. **(C)** Long exposure photos and **(D)** ML spectra (normalized) obtained under the same force of 30 N. **(E)** Integral ML intensity of ML spectra obtained under 30 N for all SrZn_2_S_2_O:Mn^2+^ samples and under varied forces for 3% Mn^2+^ doped SrZn_2_S_2_O. **(F)** CIE coordinate (x, y) values obtained from the ML spectra.

Besides UV light and X-ray energy, the potential of SrZn_2_S_2_O:Mn^2+^ to convert mechanical energy to orange light emission was reported recently by Rong-Jun Xie's group (Chen et al., 2020). As an important aspect of this multiple-energy conversion materials, the mechanoluminscence (ML) in SrZn_2_S_2_O:Mn^2+^ was further investigated herein. We fabricated the ML films containing SrZn_2_S_2_O:Mn^2+^ ML powders for ML tests with a structure depicted in Figure 5A (the fabrication process and the test detail are described in the *Experimental*). Like ZnS:Mn^2+^ and CaZnOS:Mn^2+^ ML phosphors (Chandra et al., 2013; Tu et al., 2016), the ML of SrZn_2_S_2_O:Mn^2+^ is also reproducible with no need for extra energy supplement. Intrinsic vacancies and doped Mn^2+^ impurities bring in trap levels in the bandgap of SrZn_2_S_2_O. When mechanical strain is applied, the inner crystal piezopotential generated by a polarization of the non-central symmetry structure tilts the conduction and valence band. The trapped carriers might be released to the tilting energy band, followed by the recombination of electron-hole pairs and energy transfer to Mn^2+^. ML is produced when the excited Mn^2+^ returns to ground state through radiative transition (^4^T_1_ → ^6^A_1_) (Figure 5B) (Du et al., 2019; Chen et al., 2020).

The Mn^2+^ concentration effects on ML properties were studied on homemade test equipment. Figure 5C shows the long exposure photos of ML when SrZn_2_S_2_O:Mn^2+^ samples were scraped with a force of 30 N (Figure 5C). Apparently, the ML brightness starts to decrease after increasing to a maximum when Mn^2+^ concentration is around 3%. The ML spectra were also obtained under 30 N. From the normalized ML spectra in Figure 5D, the redshift of the ML band moving from ~590 nm in 0.25% Mn to ~602 nm in 8% Mn is observed, which is larger than redshift in PL and XIL. Correspondingly, the CIE coordinate (x, y) moves from (0.557, 0.440) to (0.596, 0.401), as shown in Figure 5F. Noticed that severe quenching of ML happens only when the Mn^2+^-doping concentration is higher than 3% (Figure 5E), which is quite different from the behavior in emissions excited by UV light and X-ray (Supplementary Figure 5) but very similar to emission under excitation of Mn^2+^ d-d transition (PLE route 3). ML spectra of 0 Mn-doped SrZn_2_S_2_O were obtained under the same force of 30 N (Supplementary Figure 6). Pure SrZn_2_S_2_O (0 Mn) does not exhibit any ML at all under the test conditions (<50 N). Three percent of Mn^2+^-doped SrZn_2_S_2_O is further investigated with changing forces (Figure 5E). The integral ML intensity shows a good linear relationship with the acting force at the beginning. With larger acting force, a bigger slope is observed due to the increased contact area resulting from the deformation of the testing ML film. This linear response to applied mechanical stress makes SrZn_2_S_2_O:Mn^2+^ a good candidate for stress sensing applications. We achieved visualization of dynamic pressure distribution during handwriting on ML film containing 3% Mn^2+^-doped SrZn_2_S_2_O phosphors by extracting the grayscale of the recorded long-exposure image (Supplementary Figure 7 and Supplementary Video 2).

Previously, we, respectively, discussed the multiple energy conversion abilities in SrZn_2_S_2_O:Mn^2+^ to transform UV, X-ray, or mechanical energy into orange light emission. Noticeably different quenching behaviors have been observed for PL, XIL, and ML with increasing Mn^2+^-doping concentration. XIL has the lowest quenching concentration, which has a similar trend with PL under host lattice excitation (PLE route 1). ML has the highest quenching concentration, which is more similar to PL under direct d-d excitation (PLE route 3). It is supposed that, to some extent, the ML performance of SrZn_2_S_2_O:Mn^2+^ benefits from trap levels introduced by Mn^2+^ doping, and this is quite different from the situation in CaZnOS:Mn^2+^ reported by Zhang et al. (2015). PL under metal-to-ligand charge transfer excitation (PLE route 2) has the mediate quenching concentration.

The average distance between two neighboring Mn^2+^ in SrZn_2_S_2_O lattice decreases with increasing Mn^2+^-doping concentration resulting in the formation of more and more Mn^2+^ pairs. Due to the exchange interaction effect, the energy difference between the ground state and the first excited state reduces when Mn^2+^ pairs are formed, leading to a redshift of emission band observed in all three energy conversion modes (Barthou, 1994; Vink et al., 2001; Zhang et al., 2015). Further, the differences in XIL, PL, and ML color with the same concentration as well as their color ranges manipulated by Mn^2+^ concentration effect (Supplementary Figure 8) could be explained by the preferences in Mn^2+^ doping concentration. Take the case of ML who prefers high Mn^2+^ concentration; paired Mn^2+^ will always emit a larger proportion of longer wavelength photons, which makes ML more reddish than PL and XIL. As Mn^2+^ concentration increases, more Mn^2+^ pairs will form in SrZn_2_S_2_O lattice, and they emit an even larger proportion of longer wavelength photons leading to more redshift. The study of concentration quenching behaviors offers a better understanding of multiple energy conversion mechanisms in SrZn_2_S_2_O:Mn^2+^.

## Conclusion

In conclusion, we have presented Mn^2+^-doped SrZn_2_S_2_O crystals that display multimode energy conversion by turning X-ray, ultraviolet, and mechanical force energy into orange visible light energy via Mn^2+^ emission from ^4^T_1_ → ^6^A_1_ transition. The varied excitation mode in XIL, PL, and ML leads to performance sensitivity to doped Mn^2+^ impurities. By controlling Mn^2+^ concentration, we have obtained the most intense XIL with relatively low Mn^2+^ concentration and ML with much higher Mn^2+^ concentration, which both have a linear response to the corresponding excitation energy. The redshift of emission spectra is observed in luminescence from all three conversion modes, and the different preferences in Mn^2+^ impurities are believed to be responsible for the range of color change manipulated by Mn^2+^ doping concentration. This SrZn_2_S_2_O:Mn^2+^ with the ability of multimode energy conversion may find its application in X-ray, UV, and mechanical stress sensing and detection and multiple energy driving light sources and displays.

## Data Availability Statement

All datasets presented in this study are included in the article/Supplementary Material.

## Author Contributions

RM and DP conceived the study, designed the experiments, and wrote the manuscript. RM, SM, CW, ZW, YW, SQ, and DP carried out the material synthesis, characterization, and measurements. RM, YS, and DP analyzed the data. All authors contributed to the article and approved the submitted version.

## Conflict of Interest

The authors declare that the research was conducted in the absence of any commercial or financial relationships that could be construed as a potential conflict of interest.

## Supplementary Material

Supplementary Video 1 X-ray induced luminescence of 0.25% Mn^2+^ doped SrZn_2_S_2_O.

Supplementary Video 2 Mechanoluminescence of the 3% Mn^2+^ doped SrZn_2_S_2_O.
