# Corrigendum: Luminescence in Manganese (II)-Doped SrZn_2_S_2_O Crystals From Multiple Energy Conversion

**DOI:** 10.3389/fchem.2020.639045

**Published:** 2021-01-05

**Authors:** Ronghua Ma, Shaohui Mao, Chunfeng Wang, Yonghong Shao, Zhihao Wang, Yu Wang, Sicen Qu, Dengfeng Peng

**Affiliations:** ^1^Key Laboratory of Optoelectronic Devices and Systems of Ministry of Education and Guangdong Province, College of Physics and Optoelectronic Engineering, Shenzhen University, Shenzhen, China; ^2^International Collaborative Laboratory of 2D Materials for Optoelectronics Science and Technology of Ministry of Education, SZU–NUS Collaborative Innovation Center for Optoelectronic Science & Technology, Institute of Microscale Optoelectronics, Shenzhen University, Shenzhen, China; ^3^Department of Physical Education, Shenzhen University, Shenzhen, China

**Keywords:** light emission, mechanoluminescence, multimode luminescence, X-ray, SrZn_2_S_2_O

In the published article, there was an error in affiliation **1**. Instead of “***School of Physics and*** ***Optoelectronic Engineering, Shenzhen University, Shenzhen, China*,**” it should be “***Key*** ***Laboratory of Optoelectronic Devices and Systems of Ministry of Education and*** ***Guangdong Province, College of Physics and Optoelectronic Engineering, Shenzhen*** ***University, Shenzhen, China*.**”

The authors apologize for this error and state that this does not change the scientific conclusions of the article in any way. The original article has been updated.

