# Supplementary material for: Luminescence in Manganese (II)-Doped SrZn2S2O Crystals From Multiple Energy Conversion
Source: Front Chem. 2020 Sep 4;8:752. doi: 10.3389/fchem.2020.00752 (PMC7500203; doi:10.3389/fchem.2020.00752)
Supplement: Supplementary file 3 [file Data_Sheet_1.pdf]

Supplementary Material

Luminescence in Manganese (II) doped $SrZn_2S_2O$ Crystals from Multiple Energy Conversion

Ronghua Ma[1], Shaohui Mao[1], Chunfeng Wang[1], Yonghong Shao[1],Zhihao Wang[2],Yu Wang[2], Sicen Qu[3], Dengfeng Peng[1]*

[1]School of physics and Optoelectronic Engineering, Shenzhen University, Shenzhen 518060, China

[2]SZU–NUS Collaborative Innovation Center for Optoelectronic Science & Technology International Collaborative Laboratory of 2D Materials for Optoelectronics Science and Technology of Ministry of Education Institute of Microscale Optoelectronics, Shenzhen University, Shenzhen 518060, China

[3]Department of Physical Education, Shenzhen University, Shenzhen 518060, China

*** Correspondence:** Dengfeng Peng, E-mail: pengdengfeng@szu.edu.cn

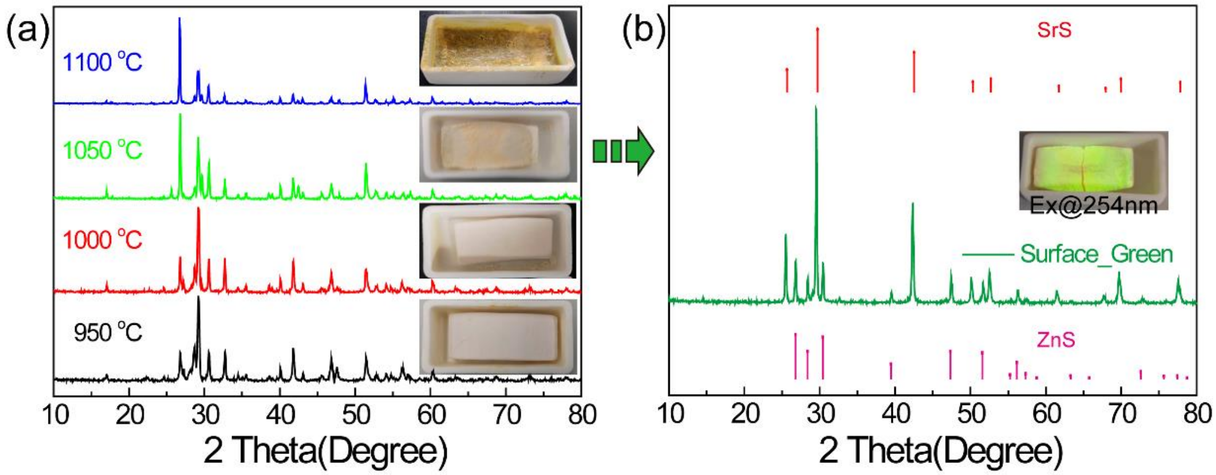

Supplementary Figure 1. (a) XRD patterns of samples obtained by annealing $SrCO_3$ and ZnS (molar ratio of 1:2) at 950, 1000, 1050, 1100 °C for 4 h. the inset images show the sample status after calcination at corresponding temperature. **(b)** XRD patterns of the surface materials collected carefully from sample annealed at 1050 °C which was green under 254 nm UV irradiation. Decomposition of $SrZn_2S_2O$ to ZnS and SrS was confirmed in 1050 °C and above, Zn and O element was supposed to be blown away in Ar flow as Zn vapor and O_2 gas.

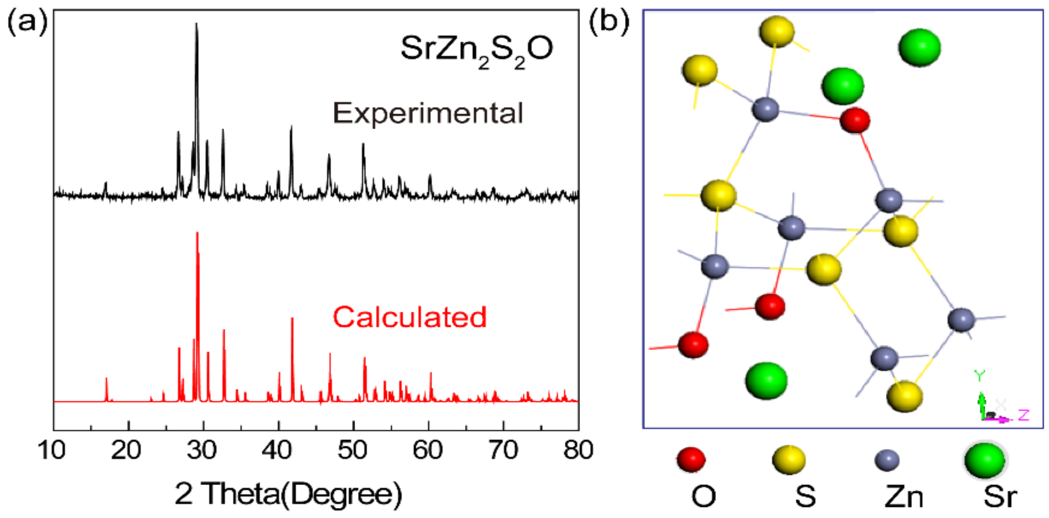

Supplementary Figure 2. (a) X-ray powder diffraction pattern of $SrZn_2S_2O$ obtained by annealing $SrCO_3$ and ZnS (molar ratio of 1:2) at 1000 °C for 4 h, the theoretical calculated pattern of $SrZn_2S_2O$ based on work of Hans-Conrad zur Loye, et al (Tsujimoto et al., 2018). **(b)** Schematic presentation of $SrZn_2S_2O$ crystal structure.

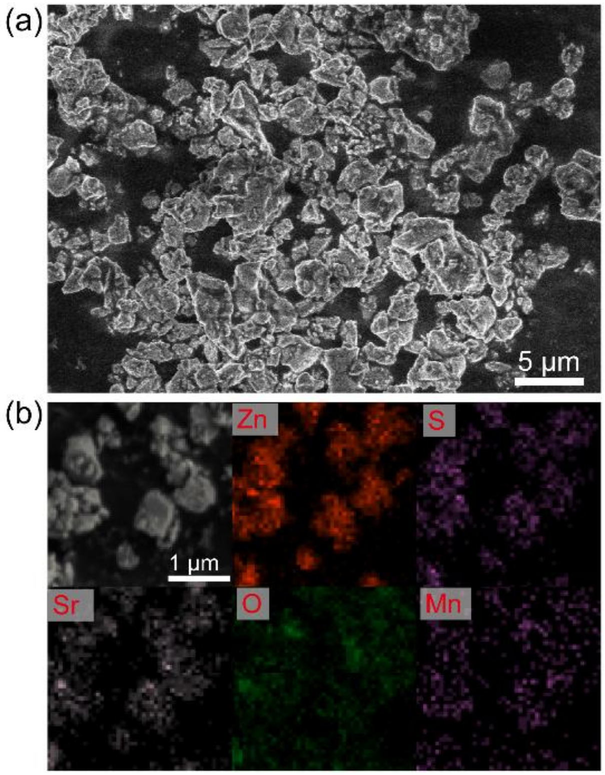

Supplementary Figure 3. (a) Scanning electron microscopy (SEM) image of $SrZn_2S_2O$ doped with 3 % Mn atoms. The powders are several micrometers in size with no regular shape **(b)** Zn, S, Sr, O and Mn element maps of $SrZn_2S_2O$ doped with 3 % Mn atoms. Uniform distribution of all elements is confirmed.

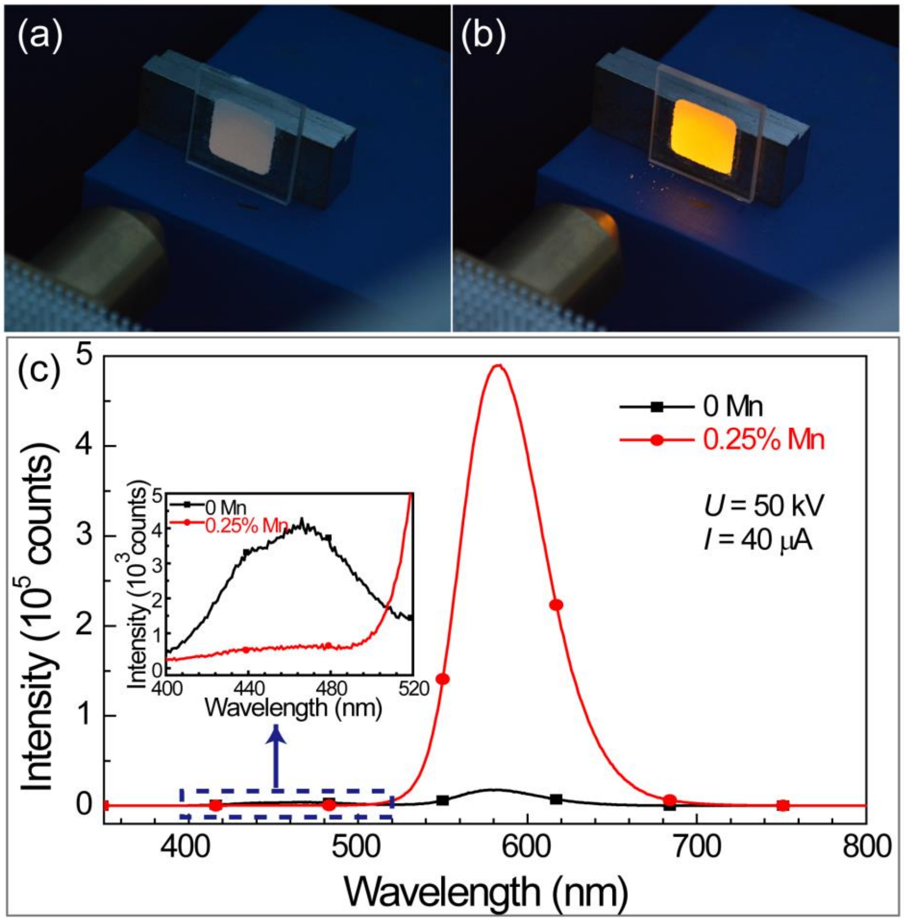

Supplementary Figure 4. X-ray induced luminescence in SrZn2S2O without doping or doped with 0.25% Mn2+. (a) the photograph of the sample that SrZn2S2O without Mn doping, showing no obvious luminescence, indicating that Mn ions plays as the effective activators in SrZn2S2O matrix (b) the photograph of the sample SrZn2S2O: Mn directly irradiated under X-ray generator (50kv, 40uA), showing obvious orange luminescence. (c) The corresponding XIL spectra in with the same conditions as in (a) and (b). Pure SrZn2S2O exhibits weak blue band due to luminescence from intrinsic defects and the weak orange luminescence is produced by trace amount of Mn impurities bring in by the raw materials while with 0.25% Mn2+ doping the blue band is suppressed and intense Mn2+ is detected.

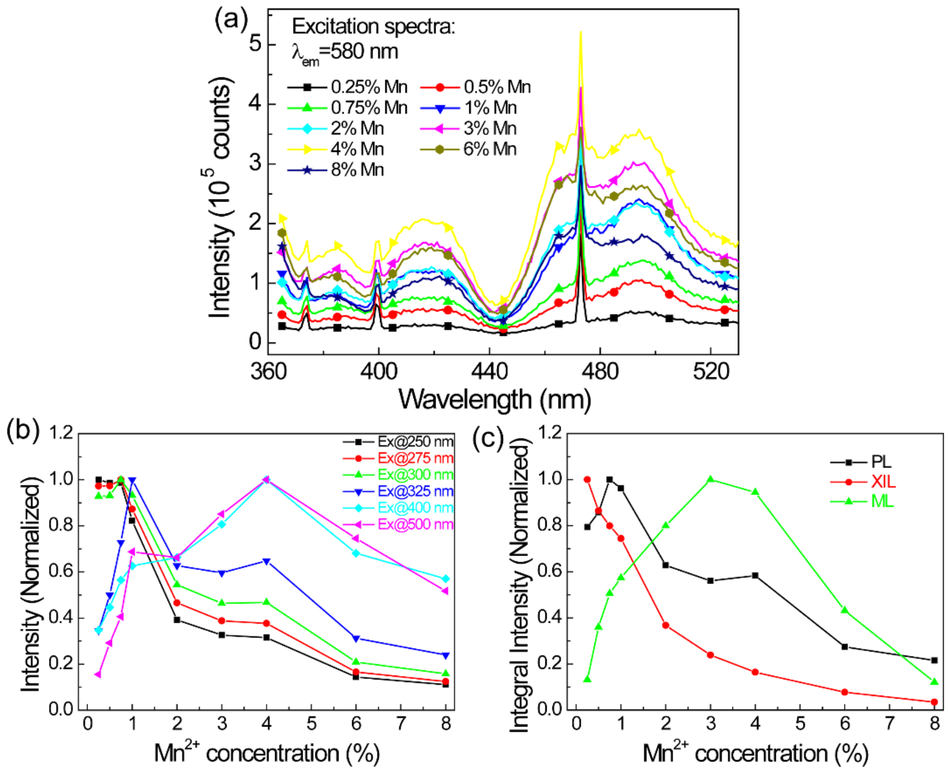

Supplementary Figure 5. (a) Enlargement of PLE spectra in **Figure 2b** to show the detail when Mn^{2+} is directly excited (PLE route 3). **(b)** PLE intensity in relation to Mn^{2+} concentration obtained in **Figure 2b** at selected wavelength of 250nm, 275 nm, 300 nm, 325 nm,400 nm and 500 nm. **(c)** Integral intensity of PL, XIL and ML in relation to Mn^{2+} concentration. Similarities trend with PLE spectra might be found.

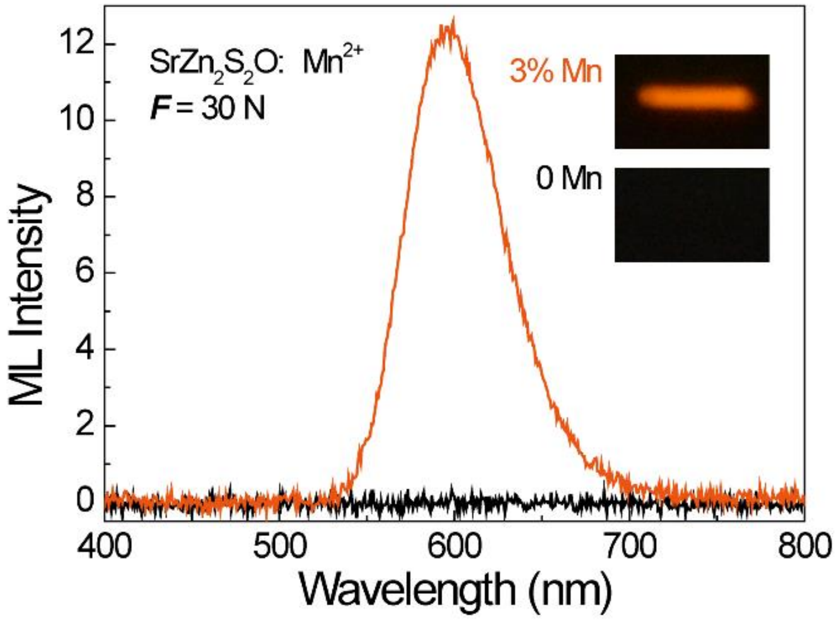

Supplementary Figure 6. ML spectra of 0 Mn and 3% Mn doped $SrZn_2S_2O$ obtained under the same force of 30 N. The inset photos are taken under the same acting force. Pure $SrZn_2S_2O$ does not exhibit any ML at all.

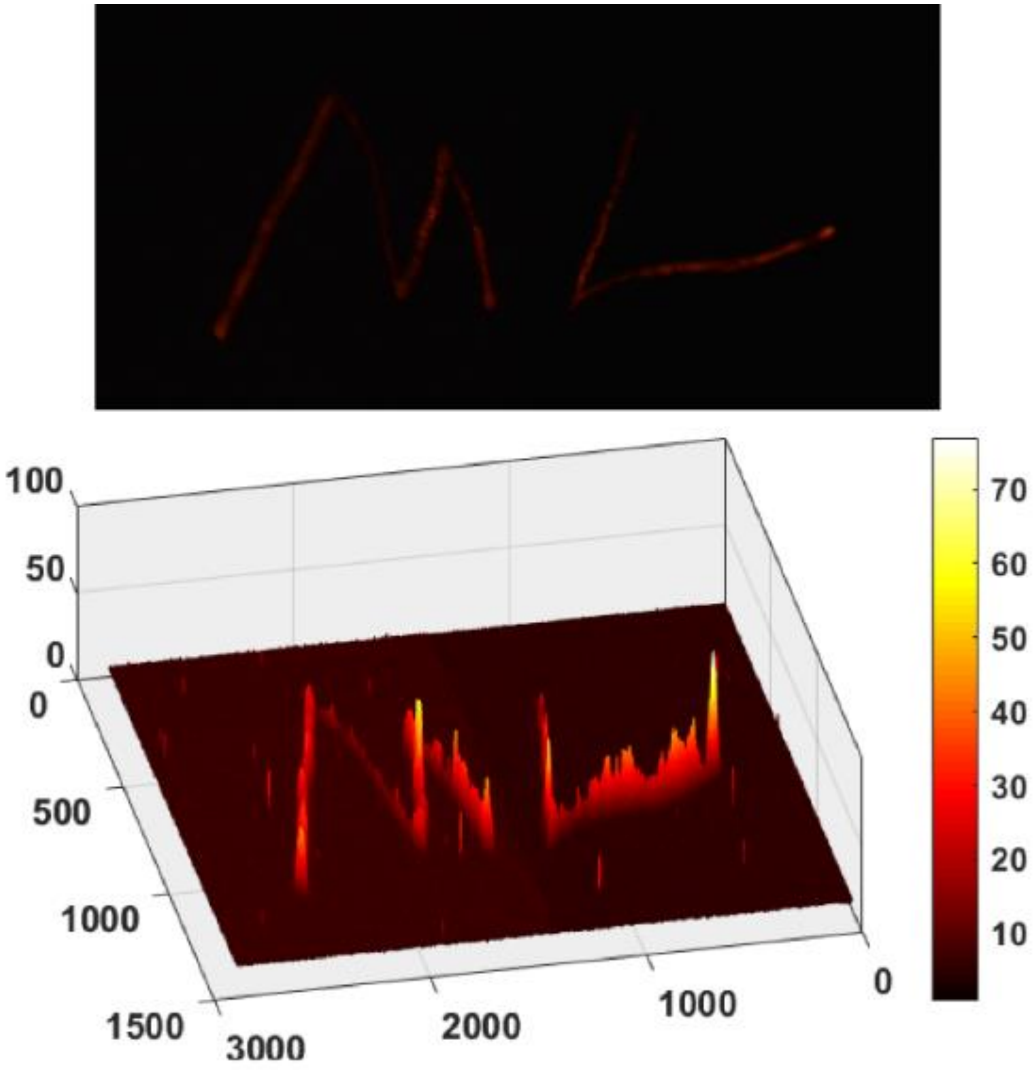

Supplementary Figure 7. Visualization of dynamic pressure distributions. Handwriting recorded by camera with long exposure and the corresponding ML intensity distribution derived from gray scale of the image.

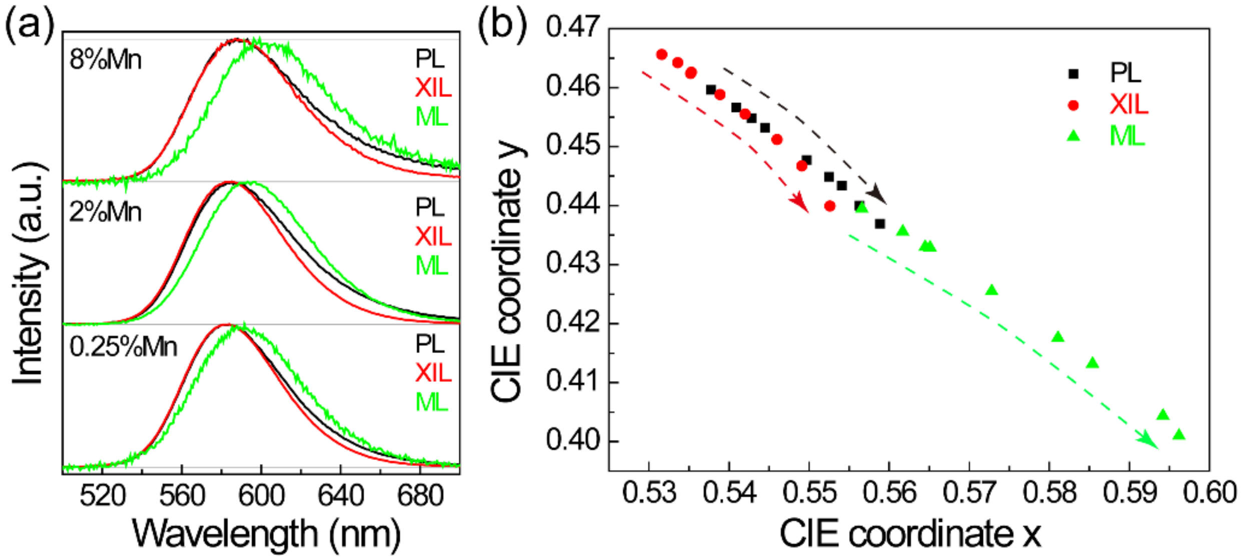

Supplementary Figure 8. (a) A comparison of normalized PL, XIL and ML spectra for 0.25%, 2% and 8% Mn^{2+} doped $SrZn_2S_2O$. **(b)** The CIE coordinate (x, y) values obtained from the tested spectra. More remarkable redshift occurs in ML.

References

Tsujimoto, Y., Juillerat, C.A., Zhang, W., Fujii, K., Yashima, M., Halasyamani, P.S., et al. (2018). Function of Tetrahedral ZnS3O Building Blocks in the Formation of SrZn2S2O: A Phase Matchable Polar Oxysulfide with a Large Second Harmonic Generation Response. *Chemistry of Materials* 30(18)**,** 6486-6493. doi: 10.1021/acs.chemmater.8b02967.
